# Impact of the COVID-19 Lockdown in Malaysia: An Examination of the Psychological Well-Being of Parent-Child Dyads and Child Behavior in Families With Children on the Autism Spectrum

**DOI:** 10.3389/fpsyt.2021.733905

**Published:** 2021-10-14

**Authors:** Hui Xian Fong, Kim Cornish, Hannah Kirk, Kartini Ilias, Mohd Farooq Shaikh, Karen Jennifer Golden

**Affiliations:** ^1^Department of Psychology, Jeffrey Cheah School of Medicine and Health Sciences, Monash University Malaysia, Subang Jaya, Malaysia; ^2^School of Psychological Sciences and Turner Institute for Brain and Mental Health, Faculty of Medicine, Nursing, and Health Sciences, Monash University, Clayton, VIC, Australia; ^3^Department of Basic Sciences, Faculty of Health Sciences, Universiti Teknologi MARA, Puncak Alam, Malaysia; ^4^Neuropharmacology Research Strength, Jeffrey Cheah School of Medicine and Health Sciences, Monash University Malaysia, Subang Jaya, Malaysia

**Keywords:** autism (ASD), COVID-19, Malaysia, lockdown, inattention, hyperactivity/impulsivity, psychological distress

## Abstract

**Background:** The COVID-19 pandemic lockdowns have adversely impacted children on the autism spectrum and their families, especially in Malaysia where this population is often marginalized. The current quantitative research aimed to investigate the impact of the Malaysian COVID-19 lockdown on the behavior and psychological distress of children formally diagnosed with an autism spectrum condition (ASC) as well as the psychological distress and well-being of their parents, in comparison with a typically developing (TD) control group.

**Methods:** The children's ages ranged between 5 and 17 years. The sample included 72 ASC parent-child dyads and 62 TD parent-child dyads. The primary caregiver completed an online survey including the following: demographic and diagnostic information; ASC symptoms; children's inattention, hyperactivity/impulsivity, perceived stress, depression, and anxiety; parents' perceived stress, depression, anxiety, and well-being based on their experience pre- and mid-lockdown (March 18th to June 9th 2020) in Malaysia.

**Results:** Among the ASC group, no significant pre- and mid-lockdown change was found in ASC symptoms (*p* > 0.05). There were no significant gender differences (boys/girls) in all the child scales. The 2 [diagnosis (ASC, TD)] × 2 [lockdown (pre-lockdown, mid-lockdown)] mixed-model ANOVAs revealed main effects of lockdown on children's attention, hyperactivity/impulsivity, anxiety, and parents' perceived stress, depression, and psychological well-being (*p* < 0.005). There was a main effect of diagnosis in all child and parent variables, except parents' perceived stress (*p* >0.005). However, there was no significant interaction effect between diagnosis and lockdown (*p* >0.005). All child behavior (inattention and hyperactivity/impulsivity) and child psychological distress (anxiety, depression, and perceived stress) were significantly correlated in both the ASC and TD groups (*p* < 0.005). On the other hand, only some of the parent variables were significantly correlated with child variables (*p* < 0.0045) in the ASC group while none of the parent variables were significantly correlated with the child variables (*p* > 0.005) in the TD group.

**Conclusion:** The results provide preliminary evidence indicating negative effects of the Malaysian lockdown on both children on the autism spectrum and TD children, as well as their parents. These quantitative results will be triangulated with the qualitative interview data to provide a holistic understanding of the impact of the pandemic, informing translational policy and practice recommendations.

## Introduction

In December 2019, the novel coronavirus, COVID-19, was discovered in China. In the face of the rapid spread of the highly infectious virus, the World Health Organization (WHO) declared it a pandemic on 11th March 2020. To combat the outbreak, the Malaysian government implemented the first nationwide Movement Control Order (MCO) from 18th March to 12th May 2020 (1–4). Restrictions such as the prohibition of mass gathering, school closures, and prohibition of outside movement other than purchasing necessities such as food or medicine were implemented during the MCO (5). On 13th May, the MCO was replaced by the Conditional MCO (CMCO) to 9th June 2020 (6), where the national economy was reopened in a controlled manner. During the CMCO, essential shops were given government approval to open for business and most people were allowed to go back to work under strict standard operating procedures (SOPs). However, the education sector, including kindergarten, government schools, and special needs schools remained closed in Malaysia (7).

Autism spectrum condition (ASC) is a heterogeneous life-long neurodevelopmental condition denoted by impairment in social interactions and communication as well as restricted and repetitive behaviors that typically appear during early childhood (8). Other than the two core symptoms, ASC is often comorbid with symptoms of other psychological and medical conditions, such as intellectual disability and attention-deficit/hyperactivity disorder [ADHD; (8, 9)]. Although there is no official prevalence statistics of ASC in Malaysia, there is evidence of a rise in the number of ASC cases seeking support over the past years. For instance, the number of intakes in special needs schools have doubled from the year 2006 to 2013 (10, 11). Moreover, doctors, psychologists, and psychiatrics have also reported an increase in the number of children on the autism spectrum in their clinics (11). These observations are urgent reminders for the need for extensive research to support this growing group.

Even though the MCO measures are essential in preventing further spreading of COVID-19, this prolonged home confinement may affect people's well-being, especially children's and adolescents' well-being. In the case of children on the autism spectrum who tend to thrive on consistent structures and patterns (8), they are particularly vulnerable in this complex situation, both from the threat of getting infected with COVID-19 (12) as well as the adverse physical and psychological impacts of the changes in environment due to the pandemic (68). For example, research in Turkey revealed elevated ASC symptoms and more problematic behaviors such as hypersensitivity, deterioration in communication, hyperactivity, and aggression among children on the autism spectrum during the home confinement period due to the disruption of their routines (13, 14). Amidst the pandemic, parents in Italy also reported that younger children on the autism spectrum were increasingly uncooperative, demanding high levels of undivided attention, and were displaying high levels of stereotypical and problematic behaviors during the lockdown (15).

For students on the autism spectrum, lockdown measures and school closures also resulted in reduced access to the resources they usually have through schools, serving as a major disruption to their daily routines (16). Although some early intervention centers in Malaysia offered online therapies during the lockdown, these services were not available to many families, either due to the lack of digital access (63, 65) or the lack of supervision of the caregivers (17). The long-term stalling of training and therapies may lead to development regression or the loss of skills that the child has acquired (18). For instance, a study in Italy illustrated the importance of school support as children on the autism spectrum who did not receive school support during home confinement displayed more severe behavioral problems than those who received regular support (19).

The home confinement and school closures may also create higher stress for children on the autism spectrum, who have high prevalence of psychological comorbidity (20, 21). For instance, the chairwoman of the National Autism Society of Malaysia (NASOM), Feilina, reported some children on the autism spectrum were more easily upset, some displayed self-injury behaviors, and even broke furniture as a result of frustration during the lockdown (22). Moreover, they may be fixated about the pandemic becoming subsequently overwhelmed by the COVID-19 information (23). For instance, individuals on the autism spectrum and their families reported experiencing higher levels of stress since the onset of the COVID-19 pandemic (24). However, some studies reported contradictory findings where children on the autism spectrum did not display any change in behaviors and psychological outcomes during the lockdown (25), or even displayed marginal improvement in emotional moods and psychopathological dimensions (68) during the pandemic. Individuals on the autism spectrum may be more comfortable at home without the academic and social requirements in school, thus having lower levels of stress during home confinement (17, 26, 64).

The pandemic and nationwide lockdown may also affect the caregivers of children on the autism spectrum disproportionately (18, 64). These unique stressors include financial constraints, disruption of carefully developed routines, disintegrated support networks, reconciling the demands of distant working and household needs, as well as the expectations to fulfill child therapies that require special training (15, 17, 27, 64). Since most of the children on the autism spectrum do not fully understand about the pandemic situation as well as about how to adapt to the new measures of social distancing and to perform proper hygiene performances (13), parents of these children experience additional stress as they have to look after their child and make sure they are safe. With the accumulated stressors mentioned above, parents of children with neurodevelopmental disabilities [NDD; (28)] as well as children on the autism spectrum (13, 68) have been reporting higher levels of anxiety and stress since the onset of the pandemic. Specifically, the parents of children on the autism spectrum reported significantly higher levels of depression and anxiety than parents of typically developing (TD) children during the lockdown (18). Parent's distress can have an adverse impact on children's behaviors and psychological well-being. In a sample of children on the autism spectrum, parent's anxiety level during the lockdown was significantly correlated with the children's mid-pandemic ASC symptoms and behavioral problems (13).

This is the first time that a quarantine to control and restrict movement has been implemented in most countries. Hence, there is a lack of conclusive studies providing data on how this quarantine can affect children and adolescents. Under usual circumstances, we might expect to see an increase in challenging behavior and skill loss in children on the autism spectrum during the holiday months, but not to the point where it cannot be addressed within the first few weeks of resuming the typical school and home intervention schedule (15). Therefore, identifying those who are at high risk and providing tailored support should be prioritized for education, health, and social care (64).

In addition, there is a need to monitor children's behavior over the long term to study how prolonged school closures, strict social distancing measures, and the pandemic itself affect the well-being of children and adolescents (29). However, most of the studies mentioned above utilized self-developed questionnaires in measuring children's outcomes and none of the study mentioned above focused on inattentive/hyperactive behaviors even though these behaviors are among the most prevalent and concerning behaviors in children on the autism spectrum (30). Furthermore, other than the study by Wang and colleagues, none of the studies mentioned above had control groups. Lastly, most of the studies mentioned originated from high-income countries, which may affect the generalizability of the findings in the context of low- and middle-income countries like Malaysia (31).

The current study aimed to investigate the change in the behaviors and psychological distress of children on the autism spectrum, as well as the psychological distress and well-being of their parents in Malaysia pre- and mid-lockdown. Based on the previous COVID-19 research, parents of children on the autism spectrum were expected to report more severe ASC symptoms mid-lockdown than pre-lockdown. Moreover, it was hypothesized that parents of children on the autism spectrum and TD children would report more behavioral problems and higher psychological distress mid-lockdown than pre-lockdown, with children on the autism spectrum displaying more behavioral problems and psychological distress than TD children. Parents of both groups were expected to report a higher level of psychological distress and lower well-being mid-lockdown than pre-lockdown, with parents of children on the autism spectrum reporting higher psychological distress and lower well-being than TD parents. The current study also aimed to explore the relationships among the children's behaviors and psychological distress as well as parents' psychological distress and well-being during the lockdown. It was hypothesized that children's behaviors and psychological distress will correlate with parents' psychological distress and well-being during the lockdown in both ASC and TD groups.

## Method

### Participants

Participants included parents who had at least one child aged between 5 and 18 years with a formal ASC diagnosis (i.e., autism spectrum disorder, autistic disorder, Asperger's disorder, and PDD-NOS) or with typical development. All families resided in Malaysia during the COVID-19 pandemic. In total, 392 parents started the survey, 133 participants were excluded due to incomplete surveys. A further 95 participants were excluded as their children were out of the age range. The final sample consisted of 134 participants, with 30 participants excluded as their children were not “typically developing” (i.e., parent-reported a child with an intellectual disability or other developmental difficulties).

The final sample comprised parent-child dyads for 72 children on the autism spectrum (54 mother-child dyads, 18 father-child dyads) and parent-child dyads for 62 TD children (53 mother-child dyads, nine father-child dyads). Demographic information for parents and children of both groups are presented in Tables 1, 2. More than half of the parents obtained at least a bachelor's degree. Almost 70% of parents are working parents. Among the working parents, almost 70% of them worked from home and around 7% of them stopped working during the MCO. During the CMCO, almost half of the working parents went back to work regularly.

**Table 1:** Parent demographics.

**Variable**	**ASC (*n* = 72)**	**TD (*n* = 62)**
Parent's age (years)	38.30 (±5.62)	37.81 (±5.93)
Household income (monthly, RM)	5,525 (±4,285)	7,570 (±5,253)
Number of people in the household	4.93 (±1.50)	4.89 (±1.12)
Number of children	2.53 (±1.19)	2.81 (±1.13)
**Relationship to child**
Mother	54 (75.0%)	53 (85.5%)
Father	18 (25.0%)	9 (14.5%)
**Ethnicity**
Malay	55 (76.4%)	47 (75.8%)
Chinese	6 (8.3%)	9 (14.5%)
Indian	5 (6.9%)	2 (3.2%)
Others	6 (8.3%)	4 (6.5%)
**State staying**
Selangor	23 (34.3%)	35 (59.3%)
KL	9 (13.4%)	8 (13.6%)
Others	40 (55.6%)	19 (30.6%)
**Education level**
Doctorate	1 (1.4%)	3 (4.8%)
Master	9 (12.5%)	9 (14.5%)
Degree	28 (38.9%)	27 (43.5%)
Diploma	15 (20.8%)	14 (22.6%)
Pre-u or foundation	7 (9.7%)	-
Secondary school	12 (16.7%)	9 (14.5%)
**Work (%)**
Working	46 (63.9%)	46 (74.2%)
Not working	26 (36.1%)	16 (25.8%)
**Among working parent - MCO work status**
Regular working	13 (28.3%)	8 (17.4%)
Work from home	29 (63.0%)	35 (76.1%)
Stopped working	4 (8.7%)	3 (6.5%)
**Among working parent - CMCO work status**
Regular working	22 (47.8%)	19 (41.3%)
Work from home	22 (47.8%)	24 (52.2%)
Stopped working	2 (4.4%)	3 (6.5%)

*MCO, Movement Control Order; CMCO, Conditional Movement Control Order*.

**Table 2:** Child demographics.

**Variables**	**ASC (*n* = 72)**	**TD (*n* = 62)**
Child's age (month)	109.06 (±35.38)	104.68 (±33.78)
**Child's gender**
Male	54 (75.0%)	37 (59.7%)
Female	18 (25.0%)	25 (40.3%)
**ASC type**
Autism spectrum disorder	8 (11.1%)	-
Autistic disorder	49 (68.1%)	-
Asperger's syndrome	10 (13.9%)	-
PDD-NOS	2 (2.8%)	-
Others	3 (4.2%)	-
**ASC severity**
Mild	38 (51.4%)	-
Moderate	23 (31.9%)	-
Severe	4 (5.6%)	-
Unspecified	7 (9.7%)	-
**ADHD comorbidity**
Yes	14 (19.4%)	-
No	58 (80.6%)	-
**Seizure/epilepsy**
Yes	2 (2.8%)	-
No	70 (97.2%)	-
**Intellectual disability**
Yes	19 (26.4%)	-
No	52 (72.2%)	-
Unsure	1 (1.4%)	-
**Type of school**
Government school	51 (70.8%)	45 (72.6%)
Private school	10 (13.9%)	10 (16.1%)
International school	1 (1.4%)	5 (8.1%)
Chinese independent school	-	2 (3.2%)
Others	10 (13.9%)	-

*PDD-NOS, Pervasive Developmental Disorder-Not Otherwise Specified; ADHD, Attention Deficit/Hyperactivity Disorder*.

The age of the children ranged from 5 years 4 months to 17 years 0 month. All diagnoses were performed by a registered mental health professional or developmental pediatrician per parent report. The Social Responsive Scale-2 [SRS-2, (32)] was utilized to help confirm the ASC diagnosis, with *T*-scores ≥ 60 used as the inclusion criteria for the ASC group and exclusion criteria in the TD group.

Eleven of the children in the ASC group had comorbid ADHD, one child on the autism spectrum had epilepsy, one child on the autism spectrum had both ADHD and epilepsy, and three children on the autism spectrum had ADHD and learning difficulties based on the parent reports. Nineteen parents of children on the autism spectrum reported their children having intellectual disability. More than half of all the children (ASC and TD) study in government schools. More than 80% of children on the autism spectrum and half of the typically developing children attend morning schools while the other half of the typically developing children attend both morning and afternoon schools every school day. Most of all the children (ASC and TD) attended online classes during the national lockdown (MCO and CMCO). Before the lockdown, more than half of the children attended additional activities (such as tuition classes or therapy sessions) but canceled these additional activities during the lockdown.

No significant difference was found in parental age between the ASC and TD groups, *t*_(130)_ = 0.46, *p* = 0.650. Moreover, there was no significant difference in child's age between the ASC and TD groups, *t*_(131)_ = 0.732, *p* = 0.465. On the contrary, there was a significant difference in child's gender between the ASC and TD groups, $\chi_{\left(1\right)}^{2}$ = 4.332, *p* = 0.037.

### Measures

#### Parent's Demographics

Participants were asked for their relationship with the child, ethnicity, religion, level of education, employment status (pre- and mid-lockdown), and socioeconomic status.

#### Child's Demographics

Participants were asked for their child's age, gender, details of the diagnosis (if applicable), schooling arrangements (pre- and mid-lockdown), as well as time allocations for various activities (e.g., screen time, physical activities) pre- and mid-lockdown.

#### COVID-19 Related Questions

Participants completed a list of questions regarding the COVID-19 situation. This included questions about their child's and their own emotions toward the pandemic, whether they or their child was infected by the virus, whether they know people infected or deceased due to the virus, where their child obtained information about COVID-19, etc.

#### ASC Symptoms

The Social Responsiveness Scale-2 [SRS-2; (33)] is a 65-item parent or teacher-report rating scale that assesses children's social impairments in naturalistic social settings. Each item is rated on a Likert scale ranging from 0 (*never true*) to 3 (*almost always true*). The SRS-2 consists of two domains that map onto the DSM-5 criteria for ASC: *Social Communication/Interaction Index* (SCI; Social Awareness, Social Cognition, Social Communication, and Social Motivation) and *Restricted/Repetitive Behavior Index* [RRB; (34)]. Higher scores on the SRS-2 indicate greater severity of social impairment and greater characteristic autistic preoccupations. Instead of the *T*-scores, raw scores were utilized for analysis as the *T*-scores are based on the Western samples (32). Satisfactory internal consistencies were obtained in the present study, with Cronbach's alpha ranging from 0.55 to 0.92 for the SRS-2 scale, as well as its indices and subscales.

#### Children's Behaviors

The Conners' Parent Rating Scales-3 [CPRS-3; (35)] is a 108-item parent report screening instrument that assesses children's indices of oppositional behavior problems, hyperactivity behavior, cognitive and inattention problems, and impulsive behavior across the home and school settings. Each item is rated on a Likert scale ranging from 0 (*Not true at al*l) to 3 (*Very much true*). The current study focused only on the *Inattention* and *Hyperactivity/Impulsivity* content scales, *Global Index* as well as the *Anxiety* and *Depression* scales, with 38 items in total. Higher scores indicate greater problems in each content scale. The *Inattention* and *Hyperactivity/Impulsivity* content scales and *Global Index* had an internal consistency ranging between Cronbach's alpha of 0.86 to 0.92 in the current research.

#### Children's Psychological Distress

The screener items for the *Anxiety* and *Depression* subscales from the CPRS-3 were used to assess children's anxiety and depression levels. Both the anxiety screener and depression screener consist of four items each. Each item is rated on a Likert scale ranging from 0 (*Not true at all*) to 3 (*Very much true*). Lower scores indicate better mental well-being. Satisfactory internal consistencies were achieved for *Anxiety* and *Depression* subscales, with Cronbach's alpha ranging 0.68 to 0.94 for the *Anxiety* subscale and 0.65 to 0.66 for the *Depression* subscale.

The NIH Perceived Stress Scale-Child [PSSC; (62)] is a scale adapted from the Perceived Stress Scale-Adult (36). It is a 10-item parent-reported screening instrument that assesses the stress experienced by the children. Similar to the adult form used in the current study, each item was rated on a 5-point Likert scale ranging from 0 (*Never*) to 4 (*Very Often*). The total score was obtained by summing the reversed scores on the four positive items and the remaining six negative items. The total score ranges from 0 to 40, with higher scores indicating higher level of perceived stress. A good internal consistency was obtained in the current study, with Cronbach's alpha ranging from 0.76 to 0.79.

#### Parents' Psychological Distress

The Depression, Anxiety, Stress Scale [DASS-21; (37)] is a self-reported questionnaire in measuring the emotional states of depression, anxiety, and stress. The *Depression* and *Anxiety* subscales were utilized in measuring the parents' depressive and anxiety symptoms. Each of the subscales has seven items and each of the items was rated on a Likert scale ranging from 0 (*did not apply to me at all*) to 3 (*applied to me very much or most of the time*). A higher score indicates a higher level of depression or anxiety symptoms. High internal consistencies of the *Depression* and *Anxiety* subscales were achieved for parents of both groups, with a Cronbach's alpha ranging from 0.79 to 0.85 for *Anxiety* subscale and 0.88 to 0.91 for *Depression* subscale, in the present research.

The Perceived Stress Scale-Adult [PSSA; (36)] is a 10-item screening instrument that assesses the perception of stress. It is one of the most widely used psychological instruments that measures the degree to which situations in one's life are perceived as stressful (38). Specifically, the items are designed to evaluate how unpredictable, uncontrollable, and overloaded participants find their lives. To make this scale more appropriate in the current COVID-19 situation, participants rated how often they had experienced these feelings on a Likert scale ranging from 0 (*Never*) to 4 (*Very Often*) before and during the lockdown. The total score was obtained by summing the reversed scores on the four positive items as well as the remaining six negative items. Hence, the total scores range from 0 to 40, with higher scores reflecting greater overall distress. High internal consistency was obtained for PSSA, with Cronbach's alpha ranging from 0.79 to 0.82 in the present research.

#### Parents' Psychological Well-Being

The Scale of Positive and Negative Experience [SPANE; (39)] is a 12-item self-report measure that assesses participants' positive and negative experiences. Each of the items was rated on a 5-point Likert scale, ranging from 1 (*Very Rarely or Never*) to 5 (*Very Often or Always*) based on their experiences pre-and mid-lockdown. The scores were divided into positive (SPANE-P) and negative (SPANE-N) scores, with 6 items in each score. Thus, each score ranged from 6 to 30. These two scores were combined by subtracting SPANE-N from SPANE-P, resulting in the balance score ranging from −24 to 24. Satisfactory internal consistencies were achieved for SPANE, with Cronbach's alpha ranging from 0.88 to 0.91 for SPANE-P and 0.84 to 0.89 for SPANE-N.

### Procedure

Ethical approval for the present study was obtained from the Monash University Human Research Ethics committee (MUHREC; Project 24673). The researchers reached out to ASC-related organizations and schools in Malaysia through initiating contact *via* email and phone calls to center administrators (e.g., Genius Kurnia). Several organizations responded and provided written permission, agreeing to post online advertisements. On top of that, digital posters were posted in several online parent support groups (e.g., Autisme Malaysia and Autism Parents Support Group Malaysia). Parents of children on the autism spectrum also helped with recruitment and referred parents of both groups. Interested parents then clicked on the link attached in the digital posters and completed the survey online.

Due to the COVID-19 pandemic, participants (parents) were provided an online written informed consent and asked to complete an online survey through the Monash University Qualtrics site. Both the English and the Malay translated versions of the questionnaires were included in the online survey. The data collection period started from late July 2020 to late October 2020. Most of the participants completed the online survey in August 2020.

The participants were asked to complete their own as well as their child's demographics. For parents of children on the autism spectrum, they answered extra demographic questions regarding their children's diagnosis. Then, participants completed a list of questions regarding the COVID-19 situation. Next, they were asked to fill the survey asking about their child's behaviors and psychological distress, as well as their own psychological distress and well-being. Importantly, they responded to these questions relative to their lives during the lockdown (18th March to 9th June 2020), and retrospectively before the lockdown.

As a token of appreciation, a RM30 ($7 USD) e-shopping voucher or e-wallet top-up was given to the participants upon completion of the online survey. After the survey completion, participants were also offered the opportunity to participate in a qualitative semi-structured interview as this quantitative study was part of a broader mixed-methods longitudinal project examining the impact of COVID-19 on children on the autism spectrum in Malaysia.

### Data Analysis

The data was analyzed using the IBM SPSS version 25 for descriptive and inferential analysis. The data was examined for univariate outliers, normality, multicollinearity, and homoscedasticity prior to the main analysis. All univariate outliers with SD > ± 3 above the mean in each group were winsorised using the formula, X = (3 × standard deviation) + mean (40). Multivariate outliers were not removed as there was no substantive difference between the analyses with and without the multivariate outliers.

Multiple two-way mixed-model analysis of variances (MM-ANOVAs), between group [diagnosis (ASC, TD)] X within group [lockdown (before, during)], were utilized to compare the mean average of the child's behaviors (as measured by Conners'-3 *Inattention* subscale, *Hyperactivity/Impulsivity* subscale, and Conners'-3 *Global Index*), child's psychological distress (as measured by Conners'-3 *Anxiety* and *Depression* subscales as well as PSSC), and parent's psychological distress (as measured by PSSA and the *Depression* and *Anxiety* subscales from DASS-21) and well-being (as measured by SPANE). Bonferroni corrections applied to *p*-values were used to reduce the risk of type I error. Hence, the adjusted alpha value was set at 0.005 (10 comparisons: alpha = 0.05/10). Levene's test statistic was used to test the assumption of homogeneity of variance. The assumption for homogeneity of variance was violated for some of the MM-ANOVAs (*p* < 0.001). However, the analysis of variance was robust to violations of this assumption as the size of the groups was reasonably similar [i.e., ASC group/TD group = 1.16; (41)].

Pearson's correlation coefficients were performed to investigate the relationship of child and parent variables mid-lockdown. Prior to the correlation analyses, statistical assumptions such as normality, linearity, and homoscedasticity were assessed. Pearson's correlations were used to understand any relations among the mid-lockdown child and parent variables in both ASC and TD groups. Bonferroni corrections applied to *p*-values were used to reduce the risk of type I error. Hence, the adjusted alpha value for the ASC group was set at 0.0045 (11 comparisons; alpha = 0.05/11) and the adjusted alpha value for the TD group was set at 0.005 (10 comparisons; alpha = 0.05/10).

## Results

### Impact of Nationwide Lockdown

Results of COVID-19 related questions are reported in Table 3. COVID-19 positivity was not reported among the parents and their children. 11.3% of the parents reported to know someone who has been hospitalized due to COVID-19 and 2.3% of the parents reported to experience people who they know passed away due to the virus. Almost all the parents reported making changes to their daily lifestyle due to the pandemic, with more than half of them (57.25%) self-quarantining most of the time. Around 5% of all the parents experienced job loss due to the pandemic. Twenty-five percent of the parents with children on the autism spectrum and 13% of parents with typically developing children reported to have lost income due to the pandemic. Around a quarter of parents (24.7%) described their household money situation as “have to cut back” since the pandemic.

**Table 3:** COVID-19 related questions distribution.

	**Diagnostic group**	
	**ASC (%)**	**TD (%)**
**How concerned do you feel about COVID-19?**	M = 3.69	M = 3.74
	SD = 0.762	SD = 0.626
Not at all concerned (1)	2 (2.8)	-
A little concerned (2)	2 (2.8)	1 (1.6)
Moderately concerned (3)	17 (23.6)	19 (30.6)
Very concerned (4)	46 (63.9)	37 (59.7)
Extremely concerned (5)	5 (6.9)	5 (8.1)
**How concerned do you feel that your child might be infected by COVID-19?**	M = 3.85	M = 3.66
	SD = 0.850	SD = 0.867
Not at all concerned (1)	1 (1.4)	-
A little concerned (2)	4 (5.6)	9 (14.5)
Moderately concerned (3)	14 (19.4)	10 (16.1)
Very concerned (4)	39 (54.2)	36 (58.1)
Extremely concerned (5)	14 (19.4)	7 (11.3)
**Have you had any flu like symptoms (e.g., fever, dry cough, shortness of breath)?**		
Yes	4 (5.6)	-
No	68 (94.4)	62 (100)
**Have you been tested for COVID-19 by a medical doctor?**		
Yes, I was tested for COVID-19 and the results were negative	2 (2.8)	3 (4.8)
No, I was not tested for COVID-19 because I could not get a test	20 (27.8)	16 (25.8)
No, I have not tried to get a test	50 (69.4)	43 (69.4)
**Do you personally know anyone that has been hospitalized due to COVID-19?**		
Yes	7 (9.7)	8 (12.9)
No	65 (90.3)	54 (87.1)
**Do you personally know anyone that has passed away due to COVID-19?**		
Yes	1 (1.4)	2 (3.2)
No	71 (98.6)	60 (96.8)
**Have you made any changes to your daily lifestyle due to COVID-19?**		
Yes	70 (97.2)	62 (100)
No	2 (2.8)	-
**How much are you self-quarantining?**		
None of the time	2 (2.8)	-
Some of the time	15 (20.8)	18 (29.0)
Most of the time	43 (59.7)	34 (54.8)
All of the time	12 (16.7)	10 (16.1)
**Have you lost your job due to COVID-19?**		
Yes	5 (6.9)	3 (4.8)
No	67 (93.1)	59 (95.2)
**Have you been unable to go to work due to COVID-19 related work changes?**		
Yes	25 (34.7)	19 (30.6)
No	25 (34.7)	22 (35.5)
N/A	22 (30.6)	21 (33.9)
**Have you lost income due to COVID-19 related work changes?**		
Yes	18 (25.0)	8 (12.9)
No	41 (56.9)	40 (64.5)
N/A	13 (18.1)	14 (22.6)
**How would you describe the money situation in your household right now?**		
Comfortable with extra	9 (12.5)	9 (14.5)
Enough but no extra	33 (45.8)	39 (62.9)
Have to cut back	24 (33.3)	10 (16.1)
Cannot make ends meet	6 (8.3)	4 (6.5)
Since hearing about COVID-19, household conflict increased.^*^	M = 2.99	M = 2.61
	SD = 1.120	SD = 1.046
Strongly agree (5)	7 (9.7)	1 (1.6)
Somewhat agree (4)	15 (20.8)	11 (17.7)
Neither agree nor disagree (3)	28 (38.9)	25 (40.3)
Somewhat disagree (2)	14 (19.4)	13 (21.0)
Strongly disagree (1)	8 (11.1)	12 (19.4)
**Has your child had any flu like symptoms (e.g., fever, dry cough, shortness of breath)?**		
Yes	2 (2.8)	2 (3.2)
No	70 (97.2)	60 (96.8)
**Has your child been tested for COVID-19 by a medical doctor?**		
Yes, he/she was tested for COVID-19 and the results were negative	1 (1.4)	-
No, he/she was not tested for COVID-19 because he/she couldn't get a test	16 (22.2)	16 (25.8)
No, he/she has not tried to get a test	55 (76.4)	46 (74.2)
Does your child know about the COVID-19 pandemic?^**^		
Yes	50 (69.4)	62 (100)
No	22 (30.6)	-
**Where did your child learn about COVID-19?**		
Through you	28 (56.0)	38 (61.3)
Other family members	-	2 (3.2)
Media	20 (40.0)	22 (35.5)
Other	2 (4.0)	-
How concerned does your child feel about the COVID-19 pandemic?^*^	M = 2.76	M = 3.18
	SD = 1.250	SD =0.820
Not at all concerned (1)	17 (23.6)	1 (1.6)
A little concerned (2)	11 (15.3)	13 (21.0)
Moderately concerned (3)	20 (27.8)	22 (35.5)
Very concerned (4)	20 (27.8)	26 (41.9)
Extremely concerned (5)	4 (5.6)	-
**How disruptive is your child's daily routine due to the lockdowns?**	M = 2.97	M = 2.85
	SD = 888	SD =0.846
Not at all disruptive (1)	3 (4.2)	1 (1.6)
A little disruptive (2)	19 (26.4)	23 (37.1)
Moderately disruptive (3)	28 (38.9)	23 (37.1)
Very disruptive (4)	21 (29.2)	14 (22.6)
Extremely disruptive (5)	1 (1.4)	1 (1.6)
Does your child have difficulties in following the modified routines?^***^	M = 2.74	M = 2.11
	SD =0.888	SD =0.925
Not at all difficult (1)	5 (6.9)	16 (25.8)
A little difficult (2)	24 (33.3)	29 (46.8)
Moderately difficult (3)	29 (40.3)	12 (19.4)
Very difficult (4)	13 (18.1)	4 (6.5)
Extremely difficult (5)	1 (1.4)	1 (1.6)

* *p < 0.05*,

** *p < 0.01*,

*** *p < 0.001*.

Parents from both groups were equally concerned about the pandemic. However, although not statistically significant, parents of children on the autism spectrum were more concerned that their child might be infected by the virus than parents of TD children. Since hearing about the pandemic, parents in the ASC group reported to have marginally more elevated household conflict than the parents in the TD group, *t*_(132)_ = −1.983, *p* = 0.049.

Based on the parent reports, all TD children knew about the pandemic while only 69.4% of children on the autism spectrum knew about the pandemic. Moreover, TD children were significantly more concerned about the pandemic than children on the autism spectrum, *t*_(132)_ = −2.224, *p* = 0.024. The lockdown was equally disruptive to the children's daily routine. However, children on the autism spectrum experienced significantly more difficulties in following the modified routines than the TD children, *t*_(132)_ = 3.973, *p* < 0.001.

Multiple two-way MM-ANOVAs were conducted to compare the effect of diagnosis and lockdown on the change in time spent on electronic devices, time spent with parents, and time spent on physical activities. Based on the MM-ANOVA, there is a significant lockdown effect on the time children spent on electronic devices, *F*_(1,124)_ = 135.14, *p* < 0.001, $\eta_{p}^{2}$ = 0.52. However, there's no group difference in time spent on electronic devices, *F*_(1,124)_ = 0.03, *p* = 0.856, $\eta_{p}^{2}$ = 0.00. On top of that, the interaction effect between lockdown and diagnosis group was not significant, *F*_(1,124)_ = 0.02, *p* = 0.882, $\eta_{p}^{2}$ = 0.00.

Similarly, the MM-ANOVA reveals a significant lockdown effect on children's time spent with their parents, *F*_(1,120)_ = 114.32, *p* < 0.001, $\eta_{p}^{2}$ = 0.49. However, there is no significant group difference in time spent with parents. The interaction effect between lockdown and diagnosis group was significant, *F*_(1,120)_ = 6.61, *p* = 0.011, $\eta_{p}^{2}$ = 0.05.

The final MM-ANOVA shows no significant lockdown [*F*_(1,122)_ = 0.18, *p* = 0.67, $\eta_{p}^{2}$ = 0.001] and interaction [*F*_(1,122)_ = 0.29, *p* = 0.591, $\eta_{p}^{2}$ = 0.002] effects on children's time spent on physical activities. However, there is a significant diagnosis group effect in time spent on physical activities, *F*_(1,122)_ = 8.32, *p* = 0.005, $\eta_{p}^{2}$ = 0.064.

### Group Differences Between Pre- and Mid-lockdown in the ASC and TD Groups

There were no significant gender differences (boy/girls) in all the child subscales (*p* > 0.05). Among the ASC group, paired samples *t*-tests were conducted to compare the mean scores of SRS-2 and its subscales pre- and mid-lockdown. The paired samples *t*-tests revealed that there was no significant difference in the mean scores of the SRS-2 and its subscales pre- and mid-lockdown (all *p* > 0.05; see Table 4).

**Table 4:** Mean and standard deviation and paired sample *T*-test for social responsiveness scales-2 in ASC group.

**Outcome**	**Before MCO**		**During MCO**			**95% CI for mean difference**				
	***M***	***SD***	***M***	***SD***	***n***		***r***	***t***	***df***	***p*-value**
SRS-2 total	97.36	23.37	98.11	25.54	72	−2.56, 4.06	0.838^***^	0.45	71	0.652
Social awareness	12.88	3.22	12.89	3.45	72	0.39, 0.42	0.869^***^	0.07	71	0.946
Social cognition	18.21	4.88	17.79	4.72	72	−0.90, 0.26	0.869^***^	−1.10	71	0.275
Social communication	33.21	8.44	32.83	9.36	72	−1.44, 0.69	0.876^***^	−0.70	71	0.484
Social motivation	16.08	4.70	16.63	5.23	72	−0.16, 1.25	0.823^***^	1.53	71	0.130
Social communication/interaction index	80.38	18.24	80.24	19.57	72	−2.51, 2.23	0.860^***^	−0.12	71	0.907
Restricted/repetitive behavior index	16.99	6.17	17.88	7.18	72	−0.17, 1.95	0.782^***^	1.67	71	0.099

*SRS-2, Social Responsiveness Scales-2*.

*** *p < 0.001*.

Multiple two-way MM-ANOVAs were conducted to compare the effect of diagnosis and lockdown on child's behaviors and psychological distress as well as parent's psychological distress and well-being. As shown in Table 5, the interaction effect between lockdown and diagnosis was not significant for all dependent variables (*p* > 0.005).

**Table 5:** Mean and standard deviations and 2 × 2 MM-ANOVA (diagnostic groups and lockdown period) comparisons for children's behavior and psychological distress, as well as parents' psychological distress and well-being.

**Variable**	**ASC**		**TD**		**ANOVA**					
	**Pre-lockdown**	**Mid-lockdown**	**Pre-lockdown**	**Mid-lockdown**	**Diagnostic group (ASC vs. TD)**		**Time points (Pre- vs. Mid-lockdown)**		**Interaction**	
	**Mean** **±** **SD**		**Mean** **±** **SD**		***F***	*** $\eta_{p}^{2}$ ***	***F***	*** $\eta_{p}^{2}$ ***	***F***	*** $\eta_{p}^{2}$ ***
CPRS-3 Inattention *df*(1, 132)	15.43 (6.23)	17.28 (6.90)	7.07 (5.13)	7.92 (5.79)	79.78^***^	0.377	14.17^***^	0.097	1.93	0.014
CPRS-3 Hyperactivity *df*(1, 132)	19.04 (8.15)	20.94 (9.61)	9.03 (5.43)	9.65 (6.12)	72.50^***^	0.355	8.75^**^	0.062	2.30	0.017
CPRS-3Global Index *df* (1,132)	13.21 (6.00)	15.22 (6.80)	5.27 (3.78)	6.10 (4.62)	93.48^***^	0.415	15.28^***^	0.104	2.68	0.020
CPRS-3Anxiety *df*(1,132)	4.39 (2.41)	4.97 (2.66)	1.35 (1.20)	1.76 (1.72)	81.67^***^	0.382	15.96^***^	0.108	0.54	0.004
CPRS-3Depression *df*(1,132)	3.56 (2.56)	3.94 (2.56)	0.98 (1.25)	1.24 (1.58)	65.18^***^	0.331	7.64^**^	0.055	0.26	0.002
PSSC *df*(1,132)	20.94 (4.11)	21.60 (5.09)	14.84 (3.95)	15.76 (4.73)	68.32^***^	0.341	7.11^**^	0.051	0.21	0.002
PSSA *df*(1,132)	19.79 (4.58)	21.37 (5.20)	18.56 (4.12)	19.46 (4.21)	5.28^*^	0.038	9.48^**^	0.057	0.73	0.005
DASS Depression *df*(1,132)	4.81 (4.27)	5.89 (5.51)	2.45 (2.67)	3.03 (3.06)	15.75^***^	0.107	9.14^**^	0.065	0.83	0.006
DASS Anxiety *df*(1,132)	4.29 (3.40)	5.32 (2.67)	2.66 (2.69)	2.81 (2.67)	13.90^***^	0.095	7.90^**^	0.056	4.47	0.033
SPANE *df*(1, 132)	5.47 (6.87)	3.14 (8.53)	8.89 (5.05)	7.84 (5.70)	15.14^***^	0.103	10.09^**^	0.071	1.46	0.011

*CPRS-3, Conners' Parent Rating Scales-3; PSSC, Perceived Stress Scale-Child; PSSA, Perceived Stress Scale-Adult; DASS, Depression Anxiety Stress Scales; SPANE, Scale of Positive and Negative Experience*.

*** *p < 0.001 level (2-tailed)*,

** *p < 0.01 level (2-tailed)*,

* *p < 0.05 level (2-tailed)*.

On the other hand, significant main effects for diagnosis group were identified for child's behavior [CPRS-3 *Inattention* [*F*_(1,132)_ = 79.78, *p* < 0.001, $\eta_{p}^{2}$ = 0.38]; CPRS-3 *Hyperactivity/Impulsivit*y [*F*_(1,132)_ = 72.50, *p* < 0.001, $\eta_{p}^{2}$ = 0.36]; CPRS-3 *Global Index* [*F*_(1,132)_ = 93.48, *p* < 0.001, $\eta_{p}^{2}$ = 0.42]], child's psychological distress [CPRS-3 *Anxiety* [*F*_(1,132)_ = 81.67, *p* < 0.001, $\eta_{p}^{2}$ = 0.38]; CPRS *Depression* [*F*_(1,132)_ = 65.18, *p* < 0.001, $\eta_{p}^{2}$ = 0.33]; and PSSC [*F*_(1,132)_ = 68.32, *p* < 0.001, $\eta_{p}^{2}$ = 0.34]] as well as parent's psychological distress [DASS *Depression* [*F*_(1,132)_ = 15.75, *p* < 0.001, $\eta_{p}^{2}$ = 0.11]; DASS *Anxiety* [*F*_(1,132)_ = 13.90, *p* < 0.001, $\eta_{p}^{2}$ = 0.10]], and parent's well-being [SPANE [*F*_(1,132)_ = 15.14, *p* < 0.001, $\eta_{p}^{2}$ = 0.10]]. On the other hand, significant main effect for diagnosis group was not identified for PSSA, *F*_(1,132)_ = 5.28, *p* = 0.023, $\eta_{p}^{2}$ = 0.04.

Significant main effects for lockdown (pre- vs. mid-lockdown) were found for child's behavior [CPRS-3 *Inattention* [*F*_(1,132)_ = 14.17, *p* < 0.001, $\eta_{p}^{2}$ = 0.10]; CPRS-3 *Hyperactivity/Impulsivity* [*F*_(1,132)_ = 8.75, *p* = 0.004, $\eta_{p}^{2}$ = 0.06]; CPRS-3 *Global Index* [*F*_(1,132)_ = 15.28, *p* < 0.001, $\eta_{p}^{2}$ = 0.10]], child's anxiety [CPRS-3 *Anxiety* [*F*_(1,132)_ = 15.96, *p* < 0.001, $\eta_{p}^{2}$ = 0.11]], and parent's psychological distress and well-being [PSSA [*F*_(1,132)_ = 9.48, *p* = 0.003, $\eta_{p}^{2}$ = 0.06], DASS *Depression* [*F*_(1,132)_ = 9.14, *p* = 0.003, $\eta_{p}^{2}$ = 0.07]; SPANE [*F*_(1,132)_ = 10.09, *p* = 0.002, $\eta_{p}^{2}$ = 0.07]]. On the other hand, significant main effects for lockdown were marginally non-significant for CPRS-3 *Depression* [*F*_(1,132)_ = 7.64, *p* = 0.007, $\eta_{p}^{2}$ = 0.06], PSSC [*F*_(1,132)_ = 7.11, *p* = 0.009, $\eta_{p}^{2}$ = 0.05] and DASS *Anxiety* [*F*_(1,132)_ = 7.90, *p* = 0.006, $\eta_{p}^{2}$ = 0.06; see Table 5].

### Relations Between Variables Mid-lockdown

Pearson's correlations among variables mid-lockdown were conducted for the ASC and TD groups separately (see Tables 6, 7). Pearson's correlations among the ASC group revealed significant correlations (*p* < 0.0045) among all child behavior indicators (CPRS-3 *Inattention*, CPRS-3 *Hyperactivity/Impulsivity* and CPRS *Global Index*) and psychological distress indicators (CPRS-3 *Anxiety*, CPRS-3 *Depression*, and PSSC). PSSA was significantly correlated with CRPS-3 *Inattention* [*r*_(72)_ = 0.396, *p* = 0.001], CPRS-3 *Hyperactivity/Impulsivity* [*r*_(72)_ = 0.408, *p* < 0.001], CPRS-3 *Global Index* [*r*_(72)_ = 0.448, *p* < 0.001], SRS-2 [*r*_(72)_ = 0.333, *p* = 0.004], and PSSC [*r*_(72)_ = 0.503, *p* < 0.001]. Moreover, PSSA was marginally correlated with CPRS-3 *Hyperactivity/Impulsivity* [*r*_(72)_ = 0.325, *p* = 0.005], CPRS-3 *Global Index* [*r*_(72)_ = 0.318, *p* = 0.006], and SRS-2 [*r*_(72)_ = 0.323, *p* = 0.006]. DASS *Depression* was significantly correlated with PSSC [*r*_(72)_ = 0.359, *p* = 0.002], PSSA [*r*_(72)_ = 0.663, *p* < 0.001], and DASS *Anxiety* [*r*_(72)_ = 0.770, *p* < 0.001]. Furthermore, DASS *Depression* was marginally correlated with CPRS-3 *Global Index* [*r*_(72)_ = 0.320, *p* = 0.006]. DASS *Anxiety* was significantly correlated with PSSC [*r*_(72)_ = 0.351, *p* = 0.003] and PSSA [*r*_(72)_ = 0.611, *p* < 0.001]. SPANE was significantly correlated with CPRS-3 *Inattention* [*r*_(72)_ = −0.399, *p* = 0.001], CPRS-3 *Hyperactivity/Impulsivity* [*r*_(72)_ = −0.381, *p* = 0.001], CPRS-3 *Global Index* [*r*_(72)_ = −0.475, *p* < 0.001], SRS-2 [*r*_(72)_ = −0.357, *p* = 0.002], PSSC [*r*_(72)_ = −0.458, *p* < 0.001], PSSA [*r*_(72)_ = −0.770, *p* < 0.001], DASS *Anxiety* [*r*_(72)_ = −0.672, *p* < 0.001], and DASS *Depression* [*r*_(72)_ = −0.775, *p* < 0.001]. Moreover, SPANE was marginally correlated with CPRS-3 *Depression* [*r*_(72)_ = −0.326, *p* = 0.005].

**Table 6:** Correlation between mid-lockdown scores in children's behavior and psychological distress as well as parents' psychological distress and well-being (ASC group).

**Variable**	***N***	**1**	**2**	**3**	**4**	**5**	**6**	**7**	**8**	**9**	**10**	**11**
CPRS-3 Inattention	72											
CPRS-3 Hyperactivity	72	0.853^***^										
CPRS-3 Global Index	72	0.884^***^	0.881^***^									
CPRS-3 Anxiety	72	0.630^***^	0.630^***^	0.590^***^								
CPRS-3 Depression	72	0.594^***^	0.536^***^	0.569^***^	0.808^***^							
PSSC	72	0.569^***^	0.533^***^	0.656^***^	0.410^***^	0.457^***^						
SRS-2	72	0.629^***^	0.499^***^	0.592^***^	0.541^***^	0.560^**^	0.648^***^					
PSSA	72	0.396^**^	0.408^***^	0.448^***^	0.269^*^	0.289^*^	0.503^***^	0.332^***^				
DASS Depression	72	0.234^*^	0.248^*^	0.320^**^	0.237^*^	0.219	0.359^**^	0.287^*^	0.663^***^			
DASS Anxiety	72	0.260^*^	0.325^**^	0.318^**^	0.274^*^	0.169	0.351^**^	0.319^**^	0.611^***^	0.770^***^		
SPANE	72	−0.399^**^	−0.381^**^	−0.475^***^	−0.277^*^	−0.326^**^	−0.457^***^	−0.357^**^	−0.770^***^	−0.775^***^	−0.672^***^	

*CPRS-3, Conners' Parent Rating Scales-3; PSSC, Perceived Stress Scale-Child; SRS-2, Social Responsive Scale-2; PSSA, Perceived Stress Scale-Adult; DASS, Depression Anxiety Stress Scales; SPANE, Scale of Positive and Negative Experience*.

*** *p < 0.001 level (2-tailed)*,

** *p < 0.01 level (2-tailed)*,

* *p < 0.05 level (2-tailed)*.

**Table 7:** Correlation between mid-lockdown scores in children's behavior and psychological distress as well as parents' psychological distress and well-being (TD group).

**Variable**	***N***	**1**	**2**	**3**	**4**	**5**	**6**	**7**	**8**	**9**	**10**
CPRS-3 Inattention	62										
CPRS-3 Hyperactivity	62	0.644^***^									
CPRS-3 Global Index	62	0.849^***^	0.724^***^								
CPRS-3 Anxiety	62	0.633^***^	0.617^***^	0.644^***^							
CPRS-3 Depression	62	0.683^***^	0.587^***^	0.719^***^	0.717^***^						
PSSC	62	0.648^***^	0.541^***^	0.612^***^	0.515^***^	0.545^***^					
PSSA	62	0.289^*^	0.199	0.286^*^	0.290^*^	0.148	0.296^*^				
DASS Depression	62	0.204	0.289^*^	0.265^*^	0.237	0.198	0.101	0.541^***^			
DASS Anxiety	62	0.110	0.291^*^	0.131	0.229	0.148	0.016	0.487^***^	0.507^***^		
SPANE	62	−0.173	−0.137	−0.179	−0.179	−0.043	−0.152	−0.622^***^	−0.492^***^	−0.329^**^	

*CPRS-3, Conners' Parent Rating Scales-3; PSSC, Perceived Stress Scale-Child; PSSA, Perceived Stress Scale-Adult; DASS, Depression Anxiety Stress Scales; SPANE, Scale of Positive and Negative Experience*.

*** *p < 0.001 level (2-tailed)*,

** *p < 0.01 level (2-tailed)*,

* *p < 0.05 level (2-tailed)*.

Similarly, Pearson's correlations among the TD group revealed significant correlations (*p* < 0.005) among all child behavior indicators (CPRS-3 *Inattention*, CPRS-3 *Hyperactivity/Impulsivity* and CPRS *Global Index*) and psychological distress indicators (CPRS-3 *Anxiety*, CPRS-3 *Depression*, and PSSC). On the other hand, none of the parent variables (PSSA, DASS *Depression*, DASS *Anxiety*, and SPANE) was significantly correlated with the child variables (*p* > 0.005).

## Discussion

With the pandemic outbreak, governments around the world had implemented nationwide lockdowns to slow the spreading of the virus. However, this measure has adverse impact on the lives of children on the autism spectrum and their families. To the authors knowledge, this is the first study that examined the impact of the COVID-19 lockdown on the behaviors and psychological distress of children on the autism spectrum as well as their parent's psychological distress and well-being in Malaysia.

Results suggest that parents of both groups were equally concerned about the COVID-19 pandemic. Fortunately, only a small number of the participants experienced job loss due to the pandemic. In terms of household conflicts since the onset of the pandemic, parents of children on the autism spectrum reported marginal elevated household conflict than parents of TD children. Based on the parent-reports, TD children were significantly more concerned about the pandemic than children on the autism spectrum. This could be explained by the fact that not all children on the autism spectrum (<70%) knew about the pandemic, which is in line with previous evidence that children on the autism spectrum might have difficulties comprehending the full situation due to their cognitive abilities (13).

Since the lockdown in Malaysia, the majority of the children in our sample stopped receiving face-to-face education and switched to online learning. Most of them also stopped their after-school activities, such as home tuition and therapies. Even though the intervention centers were granted with government approval to operate in mid-June 2020, the precautious measures for carrying out these therapies were not standardized, which created confusion to both parents and therapists. Hence, some parents were reluctant to send their child to the centers or participate in home-based therapies in fear of infection.

Consistent with previous reports [e.g., (14, 15, 28)], parents in both groups reported that the lockdown was somewhat disruptive to their child's daily routine. However, children on the autism spectrum had significantly more difficulties in following the modified routines than their TD peers. This is supported by the parent reports from other countries stating that children on the autism spectrum are having hard times adhering to the new routines during the pandemic (13). All these findings are consistent with previous reports of the elevated vulnerability of children on the autism spectrum with disruption of routine due to their executive functioning difficulties (42).

Based on the preliminary analysis, children of both groups spent significantly more time on electronic devices and time with their parent mid-lockdown. Due to the home confinement and disruption of daily routine, parents have been reporting having increased difficulties in managing daily activities and keeping their child contented (19). Hence, children may spend longer time on electronic devices. On the other hand, children of both groups spent roughly the same time on physical activities pre- and mid-lockdown, with TD children spending more time on physical activities than children on the autism spectrum. This finding contradicted with the reports during school holiday (43) and COVID-19 studies that indicated reduction in physical activities when children are out of schools (44–46).

### Group Difference (Diagnosis × Lockdown)

In examining the first hypothesis, no significant difference was found in the severity of ASC symptoms of children on the autism spectrum pre- and mid-lockdown. This finding differs from what has been reported in other COVID-19 studies [e.g., (13, 14, 19)], where parents have been frequently reporting their children on the autism spectrum displaying more ASC symptoms during the lockdown. Based on the preliminary interviews in the broader project, some children on the autism spectrum actually enjoyed staying at home during the first 2 months of the lockdown. This may help explain why the ASC severity did not elevate during the lockdown, as the survey was distributed relatively at the early stage of the lockdown.

The second hypothesis, which states that parents of children on the autism spectrum and TD children would report more behavioral problems and higher psychological distress mid-lockdown than pre-lockdown, with children on the autism spectrum displaying more behavioral problems and psychological distress than TD children, was partially supported. Children of both diagnosis groups displayed significantly more inattention, hyperactive, and global behavioral problems as well as higher levels of anxiety during the lockdown. Parallel with our results, other COVID-19 papers also indicate elevated behavioral problems among children [including children on the autism spectrum; (13, 14, 16, 19, 44–46)]. The impact of the COVID-19 lockdown appeared to be disrupting the children's routine, which in turn may be contributing to the elevated inattentive and hyperactive behaviors as well as higher levels of psychological distress. The increase in inattention and hyperactivity symptoms might be also contributed by the impatience in performing the suggested hygiene procedures (13) or struggling to stay focused throughout the online classes at home. Moreover, these negative impacts might also be associated with the more screen time children were getting during the home confinement. Previous studies have found that children who engaged in more screen time tend to display more behavioral problems such as inattention and hyperactivity (47–49) as well as higher psychological distress (50, 69).

On the other hand, children of both groups did not display significantly more perceived stress and depressive symptoms. These findings align with papers that reported marginal improvement in emotional moods and psychopathological dimensions in individuals on the autism spectrum (68) and children with ADHD (51) during the pandemic. One of the explanations to the non-significant change in stress and depression levels might be that some children, especially some children on the autism spectrum are more comfortable at home without the academic and social requirements in school during home confinement (17, 64).

Moreover, parents of children on the autism spectrum reported their child displaying more inattention, hyperactivity, and global behavioral problems as well as higher levels of depression, anxiety, and perceived stress than the TD children, both pre- and mid-lockdown. This is in line with the literature where children on the autism spectrum have been commonly reported to display more inattentive and hyperactive behaviors (52) and higher levels of psychological distress (21, 53) than their TD peers even before the pandemic.

In addition, the third hypothesis that parents of both groups were expected to report a higher level of psychological distress and lower well-being mid-lockdown than pre-lockdown, with parents of children on the autism spectrum reporting higher psychological distress and lower well-being than TD parents, was also partially supported. Parents of children of both diagnosis groups reported experiencing marginally higher levels of perceived stress and depression as well as lower level of well-being mid-lockdown. This is supported by research that has found that caregivers of children with NDDs (including children on the autism spectrum) reported worsening of mental health as well as a heightened level of stress due to the pandemic (13, 28, 68). Based on the limited qualitative and mixed method research conducted among children on the autism spectrum and their families during the pandemic, including in the project's preliminary qualitative interview analysis, the inability to leave the house and the lack of personal space (e.g., “me time”) were some of the common themes that have been mentioned by majority of the caregivers interviewed (54, 55, 67). However, the level of anxiety did not elevate significantly for these parents during the lockdown. As the survey was distributed relatively at the early stage of the lockdowns, the parents might be still adapting to the new lockdown or even enjoying the “break time” of remote working. Based on a qualitative interview research conducted in Turkey, parents reported to appreciate the quality time they spent with their families and children during the lockdown (54). Therefore, this might help explain why the anxiety level increased but did not elevate significantly during the initial stage of the lockdown.

Furthermore, parents of children on the autism spectrum reported significantly higher levels of depression and anxiety as well as lower well-being than parents of TD children pre- and mid-lockdown. On the other hand, the perceived stress level was similar for parents of both groups. Under normal time, parents of children on the autism spectrum frequently report having higher levels of anxiety and depression than parents of TD children as well as children with other neurodevelopmental conditions (56–58, 66). The pandemic and lockdown situations are notably stressful for parents of children on the autism spectrum. With the lack of therapy and social supports during the lockdown, parents of children on the autism spectrum have to manage without external help from school and therapists.

### Relationships Among Variables Mid-lockdown

The third objective of the current study was to explore the relationships among the children's behavior and psychological distress as well as parents' psychological distress and well-being during the COVID-19 lockdown in Malaysia among both ASC and TD groups. The results of mid-lockdown correlation analyses revealed that child behavioral indicators were significantly correlated with child psychological distress indicators among both ASC and TD groups. This indicates children's psychological distress might exacerbate children's inattentive and hyperactive behaviors during the COVID-19 pandemic lockdown. Hence, it is important to also take care of the children's psychological well-being in order to address their behavioral issues.

On the other hand, child behavioral and psychological indicators were only significantly correlated with parent psychological distress and well-being among the ASC group, but not in the TD group. This implies that the transactional parent-child relationship may be stronger in the autistic parent-child dyads than the typically developing parent-child dyads. The result contradicted with studies on transactional parent-child relationship that showed similar parent-child relationships between ASC and TD groups [e.g., (59)]. This might be because during stressful events such as the COVID-19 pandemic, where children's daily routines and therapies are significantly disrupted, children on the autism spectrum experience heightened stress. Hence, they might be extra reliant on their parents. The result is in line with the previous findings where the anxiety levels of parents were significantly correlated with children's ASC symptoms and behavioral problems during the pandemic (13, 60).

There are a number of limitations that should be taken into account in interpreting the findings of the study. First, the data was collected solely online. Hence, the results may not be representative of families who do not have access to the internet or electronical devices. Second, the online survey was distributed when Malaysia was in the Recovery MCO (RMCO), where most businesses were allowed to operate, and students were allowed to go to school. Therefore, some parents might be busy adapting to the new routines and had no opportunity to complete the survey. Parents' willingness to participate in the study is notable and greatly appreciated, although some of the parents had difficulties completing as it was time consuming, leading to a relatively high number of incomplete surveys. In view in these possible selection biases, our findings may underestimate the difficulties experienced by these families during the pandemic, as those experiencing more difficulties may not have the privilege to complete the survey. Third, the participants were instructed to complete the survey based on their experiences at two time periods: during the lockdown and before the lockdown in retrospective. Though we made it really clear about the timepoints in the survey, the participants' evaluation of their previous experience may have been influenced from current experience, and such recall bias can be a limitation of this study. Lastly, future studies are recommended to analyse in more detail on the children on the autism spectrum based on comorbidities such as intellectual disability and ADHD.

Despite the limitations, the present study still provides valuable information for future research. This is the first study that provides data on the behavioral and psychological distress changes due to the COVID-19 lockdown among children on the autism spectrum in Malaysia. Some practical implications could be derived from the results. For instance, the obtained data could help governments to take the needs of children with developmental conditions, such as children on the autism spectrum, and their families into consideration when deciding the confinement rules to preserve their mental health and well-being. Moreover, professionals should be alert to the more common behavioral and psychological responses of children and parents to detect the need for intervention as early as possible. Particularly, vulnerable children, such as children on the autism spectrum should receive special attention as they have higher risk factors of displaying behavioral and psychological symptoms. Lastly, knowing the specific impact of the multistage lockdowns on the behaviors and mental health of children on the autism spectrum and their families may help professionals, such as teachers, therapists, psychologists, pediatricians, psychiatrists, public health workers, or those who work with these populations to provide tailored support. For example, considering the associations between parents' mental health and children's behaviors and mental health, these relationships imply that treatment and public health policy needs to address both child and parent concerns (61).

In conclusion, it is evident that the pandemic has created a lot of stressors which impacted on the behavior and psychological well-being of children, as well as the psychological distress and well-being of their parents. After the study has concluded, the lockdown scenario has changed multiple times, from loosening the restriction to reintroducing the lockdown and eventually loosening the restrictions again. Overtime, we follow the evolution of these participants through similar surveys at different timepoints, hoping to draw conclusions on the long-term impact of these measures on the participants' clinical and non-clinical outcomes overtime.

## Data Availability Statement

The raw data supporting the conclusions of this article will be made available by the authors.

## Ethics Statement

The studies involving human participants were reviewed and approved by the Monash University Human Research Ethics Committee (MUHREC). The participants provided their written informed consent to participate in this study.

## Author Contributions

HF collected the data with the assistance of KI, conducted the data analysis, and drafted the manuscript. MS, HK, KC, KI, and KG assisted in data analysis and interpretation of results. HF, KC, HK, KI, MS, and KG contributed to revising the manuscript. KG supervized the research project and writing. All authors contributed to the writing, study conceptualization, study design, and approved the final version for publication.

## Funding

The authors would like to acknowledge that this work was supported by Monash University Malaysia and the Jeffrey Cheah School of Medicine and Health Sciences under the Monash Malaysia NEED Grant Scheme 2020-2023 (Digital Health; Project code: MED/NEED/11-2020/001).

## Conflict of Interest

The authors declare that the research was conducted in the absence of any commercial or financial relationships that could be construed as a potential conflict of interest.

## Publisher's Note

All claims expressed in this article are solely those of the authors and do not necessarily represent those of their affiliated organizations, or those of the publisher, the editors and the reviewers. Any product that may be evaluated in this article, or claim that may be made by its manufacturer, is not guaranteed or endorsed by the publisher.

## References

[1] Bernama. Covid-19: PM Outlines Several Key Measures. Daily Express. (2020). Available online at: http://www.dailyexpress.com.my/news/148684/covid-19-pm-outlines-several-key-measures/ (accessed May 17, 2021).

[2] Bernama. MCO Period Extended to April 14-PM Muhyiddin. (2020). Available online at: https://www.bernama.com/en/general/news_covid-19.php?id=1824718 (accessed May 17, 2021).

[3] Bernama. MCO Extended Until April 28 – PM Muhyiddin. (2020). Available online at: https://www.bernama.com/en/general/news_movementorder.php?id=1830577 (accessed May 17, 2021).

[4] Povera A Harun HN. MCO Phase 4 From April 29 to May 12. New Straits Times. (2020). Available online at: https://www.nst.com.my/news/nation/2020/04/586998/mco-phase-4-april-29-may-12 (accessed May 17, 2021).

[5] Bunyan J. PM: Malaysia Under Movement Control Order From Wed Until March 31, All Shops Closed Except for Essential Services. Malay Mail. (2020). Available online at: https://www.malaymail.com/news/malaysia/2020/03/16/pm-malaysia-in-lockdown-from-wed-until-march-31-all-shops-closed-except-for/1847204 (accessed May 17, 2021).

[6] Koya Z. Conditional MCO Extended for Another Four Weeks to June 9. Star Media Group Berhad. (2020). Available online at: https://web.archive.org/web/20200510181215/https:/www.thestar.com.my/news/nation/2020/05/10/conditional-mco-extended-for-another-four-weeks-to-june-9 (accessed August 10, 2021).

[7] CodeBlue. CMCO Rules Allow Most Business, Free Movement Within States. Code Blue. (2020). Available online at: https://codeblue.galencentre.org/2020/05/04/cmco-rules-allow-most-businesses-free-movement-within-states/ (accessed August 10, 2021).

[8] American Psychiatric Association. Diagnostic and Statistical Manual of Mental Disorders. 5th ed. Washington, DC: American Psychiatric Association (2013). 10.1176/appi.books.9780890425596

[9] Cornish K Wilding J. Attention, Genes, and Developmental Disorders. New York, NY: Oxford University Press (2010). 10.1093/acprof:oso/9780195179941.001.0001

[10] Razali NM Toran H Kamaralzaman S Salleh NM Yasin MHM. Teachers' perceptions of including children with autism in a preschool. Asian Social Science. (2013) 9:261–7. 10.5539/ass.v9n12p261

[11] Toran H. Experience and challenges in setting up a model demonstration classroom for children with autism in Malaysia. Int J Educ Admin Dev. (2011) 2:37–47. Available online at: https://www.yumpu.com/en/document/view/5749708/experience-and-challenges-in-setting-up-a-model-mahasarakham-

[12] Turk MA McDermott S. The COVID-19 pandemic and people with disability. Disabil Health J. (2020) 13:100944. 10.1016/j.dhjo.2020.100944 32475803 PMC7254018

[13] Mutluer T Doenyas C Genc HA. Behavioral implications of the covid-19 process for autism spectrum disorder, and individuals' comprehension of and reactions to the pandemic conditions. Front Psychiatry. (2020) 11:561882. 10.3389/fpsyt.2020.561882 33304279 PMC7701051

[14] Türkoglu S Uçar HN Çetin FH Güler HA Tezcan ME. The relationship between chronotype, sleep, and autism symptom severity in children with ASD in COVID-19 home confinement period. Chronobiol Int. (2020) 37:1207–13. 10.1080/07420528.2020.1792485 32746638

[15] degli Espinosa F Metko A Raimondi M Impenna M Scognamiglio E. A model of support for families of children with autism living in the COVID-19 lockdown: lessons from Italy. Behav Anal Pract. (2020) 13:550–8. 10.1007/s40617-020-00438-7 32837696 PMC7266382

[16] Di Giorgio E Di Riso D Mioni G Cellini N. The interplay between mothers' and children behavioral and psychological factors during COVID-19: an Italian study. Eur Child Adolesc Psychiatr. (2020) 29:1–12. 10.1007/s00787-020-01631-3 32865654 PMC7456665

[17] Ameis SH Lai MC Mulsant BH Szatmari P. Coping, fostering resilience, and driving care innovation for autistic people and their families during the COVID-19 pandemic and beyond. Mol Autism. (2020) 11:1–9. 10.1186/s13229-020-00365-y 32698850 PMC7374665

[18] Wang L Li D Pan S Zhai J Xia W Sun C. The relationship between 2019-nCoV and psychological distress among parents of children with autism spectrum disorder. Global Health. (2021) 17:1–14. 10.1186/s12992-021-00674-8 33632259 PMC7905970

[19] Colizzi M Sironi E Antonini F Ciceri ML Bovo C Zoccante L. Psychosocial and behavioral impact of COVID-19 in autism spectrum disorder: an online parent survey. Brain Sci. (2020) 10:341. 10.3390/brainsci10060341 32503172 PMC7349059

[20] Hollocks MJ Lerh JW Magiati I Meiser-Stedman R Brugha TS. Anxiety and depression in adults with autism spectrum disorder: a systematic review and meta-analysis. Psychol Med. (2019) 49:559–72. 10.1017/S0033291718002283 30178724

[21] Lai MC Kassee C Besney R Bonato S Hull L Mandy W. Prevalence of co-occurring mental health diagnoses in the autism population: a systematic review and meta-analysis. Lancet Psychiatr. (2019) 6:819–29. 10.1016/S2215-0366(19)30289-5 31447415

[22] Tan V. New Social Norms, Disruption to Routines: Trying Times for Autistic Community as Malaysia Enters COVID-19 Recovery Phase. CNA. (2020). Available online at: https://www.channelnewsasia.com/news/asia/malaysia-covid-19-autism-adjust-new-social-norms-disrupt-routine-12835204 (accessed May 21, 2021).

[23] Courtenay K Perera B. COVID-19 and people with intellectual disability: Impacts of a pandemic. Ir J Psychol Med. (2020) 37:231–6. 10.1017/ipm.2020.45 32404232 PMC7287305

[24] Manning J Billian J Matson J Allen C Soares N. Perceptions of families of individuals with autism spectrum disorder during the COVID-19 crisis. J Autism Dev Disord. (2020) 5:1–9. 10.1007/s10803-020-04760-5 33090358 PMC7578441

[25] Guidotti M Gateau A Malvy J Bonnet-Brilhault F. Does Autism Protect Against COVID Quarantine Effects? medRxiv. (2020). 10.1101/2020.10.13.20212118

[26] Vilelas J. Autistic Spectrum Disorder in the Context of Pandemic by Covid-19: Caring for Children and Caregivers. IntechOpen (2021). Available online at: https://www.intechopen.com/online-first/75520. 10.5772/intechopen.96583

[27] Eshraghi AA Li C Alessandri M Messinger DS Eshraghi RS Mittal R. COVID-19: Overcoming the challenges faced by individuals with autism and their families. Lancet Psychiatry. (2020) 7:481–3. 10.1016/S2215-0366(20)30197-8 32445682 PMC7239613

[28] Masi A Mendoza Diaz A Tully L Azim SI Woolfenden S Efron D. Impact of the COVID-19 pandemic on the well-being of children with neurodevelopmental disabilities and their parents. J Paediatr Child Health. (2021) 57:631–6. 10.1111/jpc.15285 33426739 PMC8014782

[29] Lee J. Mental health effects of school closures during COVID-19. Lancet Child Adolesc Health. (2020) 4:421. 10.1016/S2352-4642(20)30109-7 32302537 PMC7156240

[30] Hartley SL Sikora DM. Sex differences in autism spectrum disorder: an examination of developmental functioning, autistic symptoms, and coexisting behavior problems in toddlers. J Autism Dev Disord. (2009) 39:1715–22. 10.1007/s10803-009-0810-8 19582563 PMC3590797

[31] Hossain MM Sultana A Purohit N. Mental health outcomes of quarantine and isolation for infection prevention: a systematic umbrella review of the global evidence. SSRN. (2020). 10.21203/rs.3.rs-25647/v1 32512661 PMC7644933

[32] Constantino JN Gruber CP. Social Responsiveness Scale-2 (SRS-2). Los Angeles, CA: Western Psychological Services (2012).

[33] Constantino JN. Social Responsiveness Scale. 2nd ed. Los Angeles, CA: Western Psychological Services (2013). 10.1007/978-1-4419-1698-3_296

[34] Frazier TW Ratliff KR Gruber C Zhang Y Law PA Constantino JN. Confirmatory factor analytic structure and measurement invariance of quantitative autistic traits measured by the Social Responsiveness Scale-2. Autism. (2014) 18:31–44. 10.1177/1362361313500382 24019124

[35] Conners CK. Conners 3. MHS Assessments. North Tonawanda: MHS. (2015).

[36] Cohen S Kamarck T Mermelstein R. A global measure of perceived stress. J Health Soc Behav. (1983) 24:385–96. 10.2307/2136404 6668417

[37] Lovibond PF Lovibond SH. The structure of negative emotional states: Comparison of the Depression Anxiety Stress Scales (DASS) with the Beck Depression and Anxiety Inventories. Behav Res Ther. (1995) 33:335–43. 10.1016/0005-7967(94)00075-U 7726811

[38] Cohen S Kessler RC Gordon LU. Measuring Stress: A Guide for Health and Social Scientists. New York, NY: Oxford University Press on Demand (1997).

[39] Diener E Wirtz D Tov W Kim-Prieto C Choi D-w Oishi S. New well-being measures: short scales to assess flourishing and positive and negative feelings. Soc Indicat Res. (2010) 97:143–56. 10.1007/s11205-009-9493-y

[40] Field A. Discovering Statistics Using IBM SPSS Statistics. 4th ed. London: SAGE (2013).

[41] Stevens JP. Applied Multivariate Statistics for the Social Sciences. 5th ed. New York, NY: Routledge (2012). 10.4324/9780203843130

[42] Narzisi A Muratori F Calderoni S Fabbro F Urgesi C. Neuropsychological profile in high functioning autism spectrum disorders. J Autism Dev Disord. (2013) 43:1895–909. 10.1007/s10803-012-1736-0 23224514

[43] Brazendale K Beets MW Weaver RG Pate RR Turner-McGrievy GM Kaczynski AT. Understanding differences between summer vs. school obesogenic behaviors of children: the structured days hypothesis. Int J Behav Nutr Phys Act. (2017) 14:100. 10.1186/s12966-017-0555-2 28747186 PMC5530518

[44] Brooks SK Smith LE Webster RK Weston D Woodland L Hall I. The impact of unplanned school closure on children's social contact: rapid evidence review. Eurosurveillance. (2020) 25:2000188. 10.2807/1560-7917.ES.2020.25.13.2000188 32265006 PMC7140596

[45] Orgilés M Morales A Delvecchio E Mazzeschi C Espada JP. Immediate psychological effects of the COVID-19 quarantine in youth from Italy and Spain. Front Psychol. (2020) 11:2986. 10.3389/fpsyg.2020.579038 33240167 PMC7677301

[46] Xiang YT Yang Y Li W Zhang L Zhang Q Cheung T. Timely mental health care for the 2019 novel coronavirus outbreak is urgently needed. Lancet Psychiatry. (2020) 7:228–9. 10.1016/S2215-0366(20)30046-8 32032543 PMC7128153

[47] Tamana SK Ezeugwu V Chikuma J Lefebvre DL Azad MB Moraes TJ. Screen-time is associated with inattention problems in preschoolers: results from the CHILD birth cohort study. PLoS ONE. (2019) 14:e0213995. 10.1371/journal.pone.0213995 30995220 PMC6469768

[48] Ra CK Cho J Stone MD De La Cerda J Goldenson NI Moroney E. Association of digital media use with subsequent symptoms of attention-deficit/hyperactivity disorder among adolescents. J Am Med Assoc. (2018) 320:255–63. 10.1001/jama.2018.8931 30027248 PMC6553065

[49] Westby C. Screen time and children with autism spectrum disorder. Folia Phoniatrica et Logopaedica. (2021) 73:233–40. 10.1159/000506682 32229733

[50] Baker JK Fenning RM Erath SA Baucom BR Moffitt J Howland MA. Sympathetic under-arousal and externalizing behavior problems in children with autism spectrum disorder. J Abnorm Child Psychol. (2018) 46:895–906. 10.1007/s10802-017-0332-3 28736798 PMC5783799

[51] Melegari MG Giallonardo M Sacco R Marcucci L Orecchio S Bruni O. Identifying the impact of the confinement of Covid-19 on emotional-mood and behavioural dimensions in children and adolescents with attention deficit hyperactivity disorder (ADHD). Psychiatry Res. (2021) 296:113692. 10.1016/j.psychres.2020.113692 33421841 PMC7770476

[52] Lyall K Schweitzer JB Schmidt RJ Hertz-Picciotto I Solomon M. Inattention and hyperactivity in association with autism spectrum disorders in the CHARGE study. Res Aut Spectr Disord. (2017) 35:1–12. 10.1016/j.rasd.2016.11.011 29276530 PMC5738931

[53] Mayes SD Calhoun SL Murray MJ Ahuja M Smith LA. Anxiety, depression, and irritability in children with autism relative to other neuropsychiatric disorders and typical development. Res Autism Spectr Disord. (2011) 5:474–85. 10.1016/j.rasd.2010.06.012

[54] Meral BF. Parental views of families of children with autism spectrum disorder and developmental disorders during the COVID-19 pandemic. J Aut Dev Disord. (2021) 2021:1–13. 10.1007/s10803-021-05070-0 33991289 PMC8123101

[55] Neece C McIntyre LL Fenning R. Examining the impact of COVID-19 in ethnically diverse families with young children with intellectual and developmental disabilities. J Intellect Disabil Res. (2020) 64:739–49. 10.1111/jir.12769 32808424 PMC7461180

[56] Oser TK Oser SM Parascando JA Grisolano LA Krishna KB Hale DE. Challenges and successes in raising a child with type 1 diabetes and autism spectrum disorder: mixed methods study. J Med Internet Res. (2020) 22:e17184. 10.2196/17184 32217508 PMC7301267

[57] Kuusikko-Gauffin S Pollock-Wurman R Mattila ML Jussila K Ebeling H Pauls D. Social anxiety in parents of high-functioning children with autism and Asperger syndrome. J Aut Dev Disord. (2013) 43:521–9. 10.1007/s10803-012-1581-1 22733299

[58] Van Steijn DJ Oerlemans AM Van Aken MA Buitelaar JK Rommelse NN. The reciprocal relationship of ASD, ADHD, depressive symptoms and stress in parents of children with ASD and/or ADHD. J Autism Dev Disord. (2014) 44:1064–76. 10.1007/s10803-013-1958-9 24114582

[59] Neece CL Green SA Baker BL. Parenting stress and child behavior problems: a transactional relationship across time. Am J Intellect Dev Disabil. (2012) 117:48–66. 10.1352/1944-7558-117.1.48 22264112 PMC4861150

[60] Corbett BA Muscatello RA Klemencic ME Schwartzman JM. The impact of COVID-19 on stress, anxiety, and coping in youth with and without autism and their parents. Aut Res. (2021) 1–16. 10.1002/aur.2521 33913261 PMC8237027

[61] Howlin P Magiati I Charman T. Systematic review of early intensive behavioral interventions for children with autism. Am J Intellect Dev Disabil. (2009) 114:23–41. 10.1352/2009.114:23-41 19143460

[62] National Institutes of Health and Northwestern Univeristy. NIH Toolbox Perceived Stress Survey. National Institutes of Health and Northwestern Univeristy (2006).

[63] Amar-Singh. Impact of COVID-19 on Children. Free Malaysia Today. (2021). Available online at: https://www.freemalaysiatoday.com/category/highlight/2021/01/08/impact-of-covid-19-on-children/ (accessed May 21, 2021).

[64] Asbury K Fox L Deniz E Code A Toseeb U. How is COVID-19 affecting the mental health of children with special educational needs and disabilities and their families? J Aut Dev Disord. (2021) 51:1772–80. 10.1007/s10803-020-04577-2 32737668 PMC7393330

[65] Hamid HA Khalidi JR. COVID-19 and Unequal Learning. Khazanah Research Institution. (2020). Available online at: http://www.krinstitute.org/assets/contentMS/img/template/editor/20200426_Covid_Education_v3.pdf (accessed May 21, 2021).

[66] Karst JS Van Hecke AV. Parent and family impact of autism spectrum disorders: a review and proposed model for intervention evaluation. Clin Child Fam Psychol Rev. (2012) 15:247–77. 10.1007/s10567-012-0119-6 22869324

[67] Latzer IT Leitner Y Karnieli-Miller O. Core experiences of parents of children with autism during the COVID-19 pandemic lockdown. Autism. (2021) 25:1047–59. 10.1177/1362361320984317 33435701

[68] Lugo-Marín J Gisbert-Gustemps L Setien-Ramos I Español-Martín G Ibañez-Jimenez P Forner-Puntonet M. COVID-19 pandemic effects in people with Autism Spectrum Disorder and their caregivers: evaluation of social distancing and lockdown impact on mental health and general status. Res Autism Spectr Disord. (2021) 83:101757. 10.1016/j.rasd.2021.101757 33649707 PMC7904459

[69] Twenge JM Campbell WK. Associations between screen time and lower psychological well-being among children and adolescents: evidence from a population-based study. Prev Med Rep. (2018) 12:271–83. 10.1016/j.pmedr.2018.10.003 30406005 PMC6214874

